# Distinct Roles of Myeloid‐ and Hepatocyte‐PLA2G6 Deletion in Mice With Metabolic Dysfunction‐Associated Steatotic Liver Disease

**DOI:** 10.1111/liv.70679

**Published:** 2026-05-08

**Authors:** Gang Li, Simone Staffer, Sabine Tuma‐Kellner, Uta Merle, Walee Chamulitrat

**Affiliations:** ^1^ Department of Internal Medicine IV University Hospital Heidelberg Heidelberg Germany; ^2^ Department of Gastrointestinal Surgery Union Hospital, Tongji Medical College, Huazhong University of Science and Technology Wuhan China

**Keywords:** insulin resistance, lipoprotein metabolism, liver fibrosis, phospholipid metabolism, PLA2G6

## Abstract

**Background and Aims:**

Polymorphisms of group VIA calcium‐independent phospholipase A2 (iPLA2β or PLA2G6) are associated with Type‐2 diabetes, blood lipids and inflammation. Global deficiency in iPLA2β‐null mice elicited protection against hepatic steatosis but not hepatic inflammation after high‐fat diet (HFD) feeding. We aimed to determine whether HFD‐induced phenotypes could be affected by PLA2G6 deficiency specifically in myeloid cells and hepatocytes.

**Methods:**

Male control Pla2g6^flox/flox^, myeloid‐(Pla2g6^M−/−^) and hepatocyte‐(Pla2g6^Hep−/−^) specific Pla2g6‐deficient mice were subjected to chow or HFD feeding for 6 months. The contents of phospholipids, white blood cell counts, plasma cytokines and metabolic parameters were quantified. Hepatic inflammation, lymphopoiesis and fibrosis were evaluated by histology, immunohistochemistry, Western blot and qRT‐PCR.

**Results:**

Increased levels of phospholipids were observed in bone marrow‐derived macrophages and livers from chow‐fed Pla2g6^M−/−^ and Pla2g6^Hep−/−^ mice, respectively. After HFD feeding, Pla2g6^M−/−^ mice displayed a further increase in hepatic recruitment of granulocytes and lymphocytes, plasma cytokines/lipids, liver inflammation/fibrosis as well as metabolic parameters including plasma lipoproteins, plasma/liver lipopolysaccharides, liver triglycerides/non‐esterified free fatty acids, plasma insulin/leptin and HOMA‐IR. These metabolic parameters were further increased in HFD‐fed Pla2g6^Hep−/−^ mice; however, they were protected from hepatic programmed cell death and inflammatory fibrosis with attenuation of plasma lipids and cytokines. Remarkably, these metabolic parameters were also increased in both mutants under chow.

**Conclusion:**

Myeloid‐ and hepatocyte‐PLA2G6 deficiency elicited aggravation and protection against HFD‐induced hepatic inflammation, respectively. However, PLA2G6 deficiency in both cell types exacerbated insulin resistance. PLA2G6 inactivation specifically in hepatocytes may provide a potential therapy option to alleviate diet‐induced liver inflammation.

## Key Points

Cell‐specific deletion of PLA2G6 provides mechanistic phenotypic insights seen in global deletion.

## Lay Summary

PLA2G6 regulates the remodelling of phospholipids which play an important role in cell membrane structure and functions. After chronic high dietary fat consumption, we showed that the deletion of PLA2G6 in a cell‐specific manner can lead to an opposite outcome, that is, with worsened and improved liver inflammation upon deletion in immune and liver cells, respectively. Therefore, cell‐specific regulation of PLA2G6 influences the progression of MASLD.

**Abbreviations**
AA: arachidonic acid
ACSL4: acyl‐CoA synthetase long‐chain family member 4
ALT: alanine aminotransferase
ANGPTL3: angiopoietin‐like3
AST: aspartate aminotransferase
ATG5: autophagy‐related gene5
ATGL: adipose triglyceride lipase
BMDM: bone marrow‐derived macrophages
Chol: cholesterol
COL1A1: collagen1A1
ECP: eosinophil cationic protein
ESCRT: endosomal sorting complex required for transport
FA: fatty acids
FAS: fatty acid synthase
HDL: high‐density lipoproteins
HFD: high‐fat diet
HL: hepatic lipase
HOMA‐IR: homeostasis model assessment of insulin resistance
KC/CXCL1: keratinocyte chemoattractant/C‐X‐C motif chemokine receptor 1
LC3‐I: microtubule‐associated protein light chain 3‐I
LDL: low‐density lipoproteins
LPL: lysophospholipids
LPS: lipopolysaccharides
Ly6G: lymphocyte antigen 6 family member G
MASH: metabolic‐associated steatohepatitis
MASLD: metabolic dysfunction‐associated steatotic liver disease
MBOAT: membrane‐bound O‐acyltransferase
MCD: methionine‐ and choline‐deficient
MLKL: mixed lineage kinase domain‐like protein
NAS: NAFLD activity scores
NEFA: non‐esterified free fatty acids
NOX2: NADPH oxidase 2
PC: phosphatidylcholine
PE: phosphatidylethanolamine
PI: phosphatidylinositol
PL: phospholipids
PLA2G6/iPLA2β: group VIA calcium‐independent phospholipase A2
PLIN2: perilipin2
PNPLA: patatin‐like phospholipase domain containing protein
PS: phosphatidylserine
TG: triglycerides
VLDL: very‐low‐density lipoproteins
WBC: white blood cells

## 1 Introduction

Metabolic dysfunction‐associated steatotic liver disease (MASLD) is the most common chronic liver disease globally affecting over one‐third of adults [1]. Progression to metabolic dysfunction–associated steatohepatitis (MASH) leads to the development of hepatic fibrosis, cirrhosis and hepatocellular carcinoma. In addition to high‐fat consumption, MASLD is driven by inherited and acquired genetics [2]. Genome‐wide association studies have identified 17 MASLD‐associated foci including patatin‐like phospholipase domain containing 3 (PNPLA3), PNPLA2 and membrane‐bound O‐acyltransferase 7 (MBOAT7) [3]. Moreover, two additional phospholipases (PLA2), secretory phospholipase A2 group IIA (encoded by PLA2G2A) [4] and group VIA calcium‐independent phospholipase A2 (iPLA2β or PLA2G6) [5, 6], have been associated with risks of metabolic syndrome, plasma triglycerides (TG) and Type‐2 diabetes. PLA2G6 is also among the 95 loci linked to plasma TG [7] and TG‐to‐high density lipoprotein ratio [8]. Thus, MASLD and Type‐2 diabetes risks may involve genetic variants of lipid hydrolases with specificities for TG and phospholipids (PL).

Belonging to the PNPLA protein family, PLA2G6 or PNPLA9 is a cytosol‐associated enzyme that does not require calcium for hydrolysis of PL at the sn‐2 position to generate a lysophospholipid (LPL) and a fatty acid (FA) [9]. As a result of disturbed PL metabolism [10], mutations in the PLA2G6 gene manifest as heterogeneous neurodegenerative disorders [11]. PLA2G6 polymorphisms are also associated with plasma C‐reactive protein [12] suggesting its role in systemic inflammation. Owing to its wide expression, global deletion in iPLA2β‐null mice leads to multiple abnormalities including neurological disorders, inflammation, male infertility and metabolic disorders [9]. Notably, MASLD in humans leads to severe depletion of hepatic phosphatidylcholine (PC) and phosphatidylethanolamine (PE) [13]. We have demonstrated that PL replenishment in iPLA2β‐null mice protects against obesity and hepatic steatosis induced by high‐fat diet (HFD) feeding [14]. However, iPLA2β‐null mice were not protected from HFD‐induced inflammation [14] and they even exhibited exacerbated hepatic fibrosis during nonobese MASH induced by methionine‐ and choline‐deficient (MCD) diet [15]. It was hypothesized that this exacerbation was due to activation of myeloid cells.

The role of iPLA2 on macrophage biology has been studied for nearly three decades [9, 16]. Peritoneal macrophages from iPLA2β‐null mice exhibited a shift from M1 to M2 phenotypes with upregulation of chemokine MCP‐1 [17]. We performed selective deletion of iPLA2β/PLA2G6 in myeloid cells (Pla2g6^M−/−^) and hepatocytes (Pla2g6^Hep−/−^) in order to resolve the phenotypes of iPLA2β‐null mice under MCD diet [15]. Pla2g6^M−/−^mice fed MCD diet displayed exacerbation of inflammatory MASH and their macrophages showed activation of M2/Th2 cytokines and chemokines [18]. However, Pla2g6^Hep−/−^ mice were protected from MCD‐induced MASH with downregulation of hepatic FA uptake and TG synthesis genes concomitant with an increase in cytoprotective eicosanoid lipoxin A4 in blood and liver.

Because MCD diet model of nonobese MASH does not exhibit insulin resistance [19], we aimed to investigate the phenotypes of Pla2g6^M−/−^ and Pla2g6^Hep−/−^ mice in obese model with insulin resistance by HFD feeding for 6 months [14]. In this model, Pla2g6^M−/−^ mice again displayed stark pro‐inflammatory phenotypes with hepatic inflammation, immune cell composition and fibrosis, while protection was observed in Pla2g6^Hep−/−^ mice. However, both mutants exhibited worsened plasma lipoproteins, liver lipids and HOMA‐IR, indicating distinct phenotypes from the MCD model [18]. For translational significance, selective PLA2G6 inactivation in hepatocytes may be used as a therapeutic strategy for the treatment of targeted MASLD patients who are prone to liver inflammatory fibrosis.

## 2 Methods

Materials and methods are presented as Appendix S1.

## 3 Results

### 3.1 Myeloid‐ and Hepatocyte‐PLA2G6 Deficiency Attenuates HFD‐Induced Obesity by Activating Hepatic cPLA2α Metabolism but With an Opposite Response on Plasma Lipids

**FIGURE 1:**
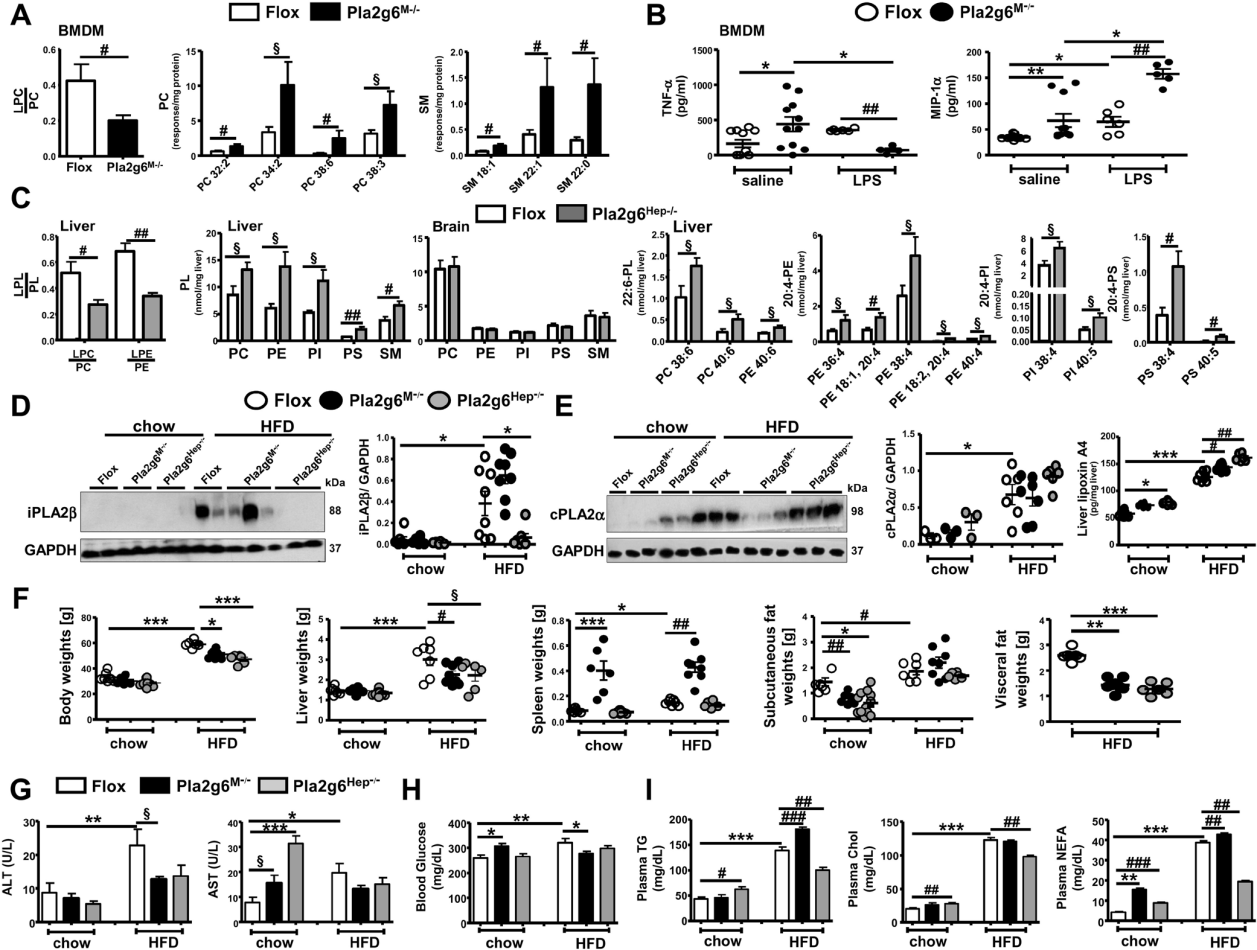
Gross phenotypic changes in body/tissue weights and blood parameters of Flox, Pla2g6^M−/−^ and Pla2g6^Hep−/−^ mice under chow or HFD. Male control (Flox), Pla2g6^M−/−^ and Pla2g6^Hep−/−^ mice at 6 months of age were fed with chow or HFD for 6 months. BMDM were prepared from male Flox and Pla2g6^M−/−^ mice treated with saline or 1 mg/kg LPS for 24 h. (A) By LC–MS/MS method, lipidomic profiles were determined in BMDM from chow‐fed Flox and Pla2g6^M−/−^ mice (*N* = 4–5). (B) The release of TNF‐α and MIP‐1α by BMDM from Flox and Pla2g6^M−/−^ mice treated with saline or LPS in vivo. (C) Left‐hand shows lipidomic profiles in liver and brain of chow‐fed Flox and Pla2g6^Hep−/−^ mice (*N* = 5–6). Right‐hand profiles showed the levels of individual PL species containing docosahexaenoic acid (22:6) and arachidonic acid (20:4) in liver. (D) Western blot analysis of hepatic iPLA2β protein (left) and quantification (right, *N* = 6–8). (E) Western blot analysis of hepatic cPLA2α protein (left) and quantification (middle, *N* = 3–7) as well as the contents of liver lipoxin A4 (pg/mg liver) as determined by ELISA (*N* = 6–13). (F) The weights (g) of body, liver, spleen, subcutaneous (inguinal) fat and visceral (retroperitoneal and perirenal) fat. (G) Plasma ALT and AST activities (U/L). (H) Blood glucose (mg/dL). (I) Plasma TG, Chol and NEFA (mg/dL). Data are mean ± SEM, *N* = 6–12 (F–I). *p < 0.05, **p < 0.01 and ***p < 0.001 by Kruskal–Wallis tests with Dunn's selected pair post‐tests; ^§^ *p* < 0.05 by one‐tailed or ^#^ *p* < 0.05, ^##^ *p* < 0.01, ^###^ *p* < 0.001 by two‐tailed Mann–Whitney *U* tests. ALT, alanine aminotransferase; AST, aspartate aminotransferase; BMDM, bone marrow‐derived macrophages; Chol, cholesterol; HFD, high‐fat diet; LC–MS/MS, liquid‐chromatography mass spectrometry; LPC, lysophosphatidylcholine; LPE, lysophosphatidylethanolamine; LPL, lysophospholipids; LPS, lipopolysaccharides; NEFA, non‐esterified fatty acids; PC, phosphatidylcholine; PE, phosphatidylethanolamine; PI, phosphatidylinositol; PL, phospholipids; PS, phosphatidylserine; TG, triglycerides; SM, sphingomyelin.

To characterize mutant phenotypes, we analysed the profiles of PL in bone marrow‐derived macrophages (BMDM) of Pla2g6^M−/−^ and livers of Pla2g6^Hep−/−^ mice under chow feeding. As the ratio of product LPL and substrate PL indicates PLA2 activity, PLA2G6 deficiency led to reduced activity seen by a decrease in LPC/PC in Pla2g6^M−/−^ BMDMs (Figure 1A) as well as LPC/PC and LPE/PE in Pla2g6^Hep−/−^ livers (Figure 1C). The levels of PC 32;2, PC 34:2, PC 38:6, PC 38:3 and sphingomyelin (SM) were increased in Pla2g6^M−/−^ BMDM (Figure 1A). This increase was associated with an elevated release of TNF‐α and MIP‐1α in saline‐treated Pla2g6^M−/−^ mice, and a shift from TNF‐α toward MIP‐1α in Pla2g6^M−/−^ mice treated with 1 mg/kg lipopolysaccharides (LPS) for 24 h (Figure 1B). In elucidating cell‐type specificity, an increase of the total PC, PE, phosphatidylinositol (PI), phosphatidylserine (PS) and SM was observed in livers but not brain tissues from Pla2g6^Hep−/−^ mice (Figure 1C). The increase of liver PC, PE, PI and PS was primarily attributable to those species containing arachidonic (20:4, AA) and docosahexaenoic (22:6) acids, consistent with the substrate preference of PLA2G6 [10].

MASLD was established by feeding male Flox control, Pla2g6^M−/−^ and Pla2g6^Hep−/−^ mice with HFD for 6 months. HFD feeding of Flox mice upregulated hepatic iPLA2β expression which was completely downregulated in Pla2g6^Hep−/−^ but not in Pla2g6^M−/−^ mice, thus confirming hepatocyte‐specific deletion of iPLA2β (Figure 1D). We previously reported that Pla2g6^M−/−^ mice challenged with LPS showed upregulated expression of hepatic cytosolic PLA2α (cPLA2α or PLA2G4a) [20]. In our cohort, HFD‐induced upregulation of cPLA2α expression was however not altered in either mutant (Figure 1E, left). Because PE and PI containing AA were increased in Pla2g6^Hep−/−^ mice (Figure 1C) indicating PLA2G6 specificity for AA [10], and these AA‐PL can be further hydrolyzed by cPLA2α to generate AA‐derived eicosanoids. We therefore analysed cytoprotective eicosanoid lipoxin A4, which was previously shown to be stepwise increased in plasma and liver of Pla2g6^Hep−/−^ mice under chow and MCD [18]. HFD feeding of Flox mice elevated hepatic levels of lipoxin A4 which were further elevated in Pla2g6^M−/−^ and Pla2g6^Hep−/−^ mice (Figure 1E, right). In our previous work [20], chow‐fed Pla2g6^M−/−^ mice displayed increased levels of liver leukotriene B4. Here Pla2g6^Hep−/−^ mice under chow and HFD also showed a stepwise increase in liver lipoxin A4. These data suggest an intrinsic role of PLA2G6 in cPLA2α metabolism and eicosanoid synthesis in myeloid cells and hepatocytes.

We determined gross phenotypic alterations of our mouse cohort. Compared to Flox counterparts, HFD‐fed Pla2g6^M−/−^ and Pla2g6^Hep−/−^ mice were protected against obesity with reduction of body and liver weights by ~13%–25% (Figure 1F). Pla2g6^M−/−^ mice displayed increased spleen weights under both diets (Figure 1F), which was previously observed in male Pla2g6^M−/−^ mice under basal conditions [20]. Both mutants showed moderate reductions in subcutaneous fat weights under chow and marked reductions in visceral fat weights under HFD (Figure 1F). HFD‐fed Pla2g6^M−/−^ and Pla2g6^Hep−/−^ mice showed a trend toward decreased ALT and AST activities, and those of ALT were significantly attenuated in Pla2g6^M−/−^ mice (Figure 1G). Remarkably, both mutants under chow showed elevated AST activities. While there were no effects on plasma glucose in Pla2g6^Hep−/−^ mice, Pla2g6^M−/−^ mice showed an increase and a decrease in plasma glucose under chow and HFD, respectively (Figure 1H). Compared to Flox counterparts, HFD‐fed Pla2g6^M−/−^ and Pla2g6^Hep−/−^ mice showed a further increase and a decrease in plasma TG and non‐esterified free fatty acids (NEFA), respectively (Figure 1I). Only Pla2g6^Hep−/−^ mice under HFD showed a decrease in plasma cholesterol (Chol). Under chow, Pla2g6^M−/−^ mice showed an increase in plasma NEFA while Pla2g6^Hep−/−^ mice showed an increase in all three plasma lipids. Despite the protection against HFD‐induced obesity, divergent effects on plasma lipids were observed with exacerbation in Pla2g6^M−/−^ mice but attenuation in Pla2g6^Hep−/−^ mice.

### 3.2 PLA2G6 Deficiency Leads to Elevated Plasma Lipoproteins, Exacerbation of Hepatic Lipids, Hyperleptinemia and Hyperinsulinemia in Pla2g6^M^ ^−/−^ and Pla2g6^Hep^ ^−/−^ Mice After HFD Feeding

**FIGURE 2:**
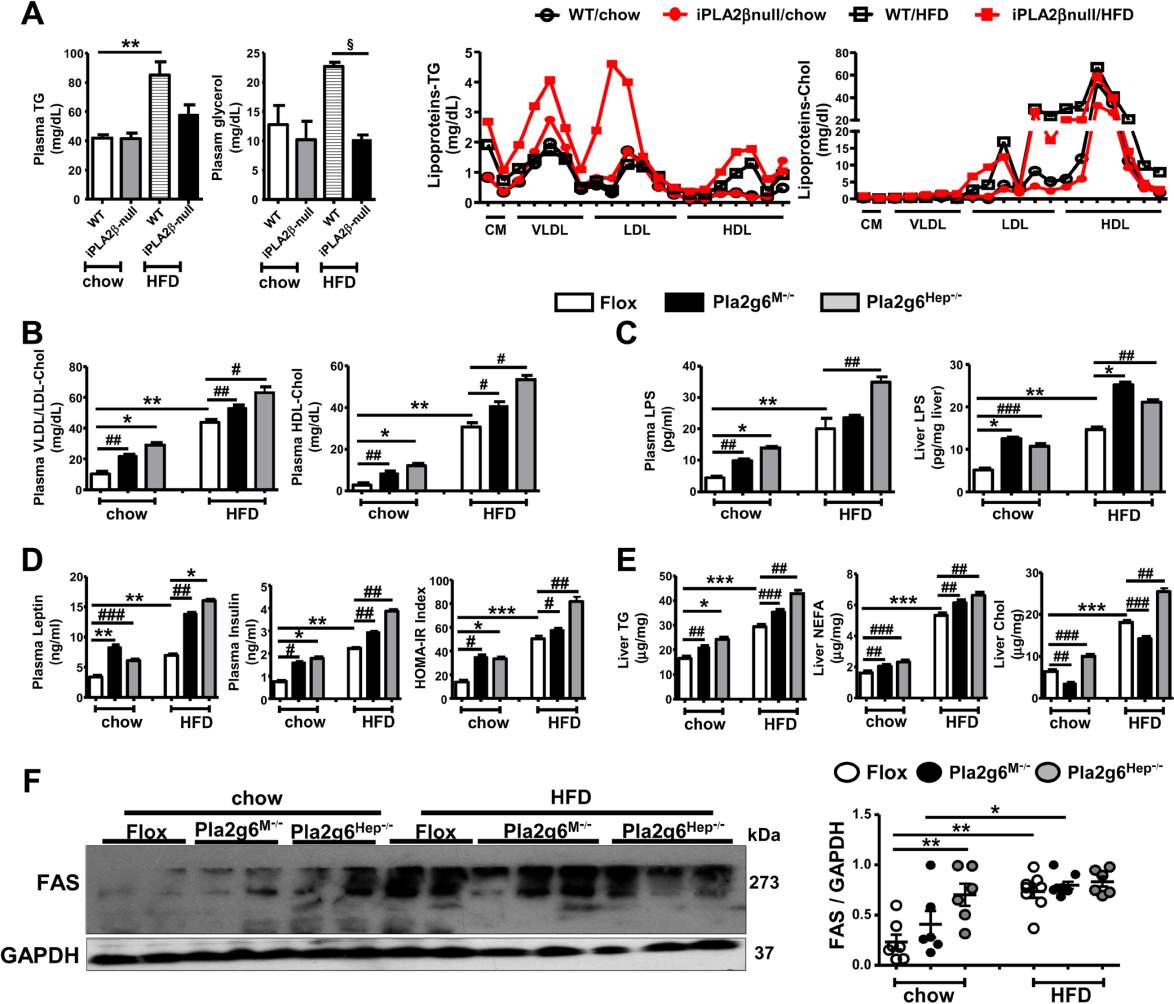
Aberrant plasma TG and lipoproteins in HFD‐fed iPLA2β‐null mice revealed divergent plasma TG and convergent plasma lipoproteins, leptin, insulin and liver TG in HFD‐fed Pla2g6^M−/−^ and Pla2g6^Hep−/−^ mice. iPLA2β‐null (C57BL/6 as WT controls), Pla2g6^M−/−^ and Pla2g6^Hep−/−^ (Flox as controls) were fed with chow or HFD for 6 months. (A) Left panel shows the levels (mg/dL) of plasma TG and glycerol (*N* = 5–12). Right panel shows the profiles of TG‐rich and Chol‐rich lipoproteins (CM, VLDL, LDL and HDL) (mg/dL) analysed by gel‐permeation high‐performance liquid chromatography in pooled samples (an average of three pooled samples from 2 to 3 mice per sample) from WT and iPLA2β‐null mice fed with chow or HFD. (B) The levels of VLDL/LDL‐Chol and HDL‐Chol (mg/dL) as determined by Sigma kits in plasma from Flox, Pla2g6^M−/−^ and Pla2g6^Hep−/−^ mice fed with chow or HFD (*N* = 4–8). (C) The levels of plasma LPS (pg/mL) and liver LPS (pg/mg liver) (*N* = 4–8). (D) The levels of plasma insulin and leptin (ng/ml) as well as HOMA‐IR index (*N* = 3–12). (E) The levels of liver TG, NEFA and Chol (μg/mg liver) (*N* = 6–12). (F) Western blot analysis of hepatic FAS protein (left) and quantification (right, *N* = 6–8). Data are mean ± SEM. *p < 0.05, **p < 0.01 and ***p < 0.001 by Kruskal–Wallis tests with Dunn's selected pair post‐tests; ^§^ *p* < 0.05 by one‐tailed or ^#^ *p* < 0.05, ^##^ *p* < 0.01, ^###^ *p* < 0.001 by two‐tailed Mann–Whitney *U* tests. Chol, cholesterol; CM, chylomicrons; FAS, fatty acid synthase; HDL, high‐density lipoproteins; HOMA‐IR, homeostatic model assessment of insulin resistance; LDL, low‐density lipoproteins; LPS, lipopolysaccharides; NEFA, non‐esterified fatty acids; TG, triglycerides; VLDL, very‐low‐density lipoproteins.

In our previous study, HFD‐fed iPLA2β‐null mice showed only a trend toward attenuation of plasma TG (Figure 2A, left), which may reflect the opposing effects of this parameter in Pla2g6^M−/−^ and Pla2g6^Hep−/−^ mice under HFD (Figure 1I). In another study using iPLA2β‐null mice with exon 2 deletion, HFD feeding of these mutants increased the secretion of VLDL and LDL from their hepatocytes in vitro [21]. We therefore examined plasma lipoprotein profiles from iPLA2β‐null mice [14] using gel‐permeation high‐performance liquid chromatography. HFD‐fed iPLA2β‐null mice showed a decrease in plasma glycerol, but an increase in plasma TG‐rich VLDL and LDL as well as Chol‐rich large‐sized LDL particles (Figure 2A). Thus, under HFD, global iPLA2β deletion in exon 2 [21] and exon 9 (Figure 2A) led to elevation of secreted VLDL/LDL without significantly altering steady‐state plasma TG.

To gain insights into lipoprotein biogenesis, we determined plasma lipoproteins from our cohort of Flox, Pla2g6^M−/−^ and Pla2g6^Hep−/−^ mice by using ELISA kits. Remarkably, the levels of VLDL/LDL‐Chol and HDL‐Chol were moderately elevated in both mutants under chow, and these levels were elevated further under HFD (Figure 2B). It is known that lipoproteins bind to LPS [22], we therefore analysed LPS levels in our cohort. Similar to lipoprotein profiles (Figure 2B), LPS levels were stepwise increased from chow to HFD in both plasma and liver of Pla2g6^M−/−^ and Pla2g6^Hep−/−^ mice (Figure 2C), suggesting that liver LPS accumulation may reflect increased lipoprotein contents.

It is also known that HFD feeding increases LPS levels known as metabolic endotoxemia [23], the elevation of liver LPS in both mutants may imply an exacerbation of hepatic steatosis. Lipoproteins in mouse livers could be visualized as lipid particles by electron microscopy and indirectly quantified as liver TG [24]. Lipoprotein‐associated PC can also be a source of liver TG [25]. We therefore measured liver TG and other lipids in lipid extracts from our mouse cohort. In parallel to lipoproteins and LPS (Figure 2B,C), the levels of liver TG and NEFA were elevated in both mutants in a stepwise manner from chow to HFD (Figure 2D). Liver Chol levels were increased in Pla2g6^Hep−/−^ but decreased in Pla2g6^M−/−^ mice under chow and HFD. Plasma leptin, insulin and homeostasis model assessment of insulin resistance (HOMA‐IR) levels were stepwise increased in both mutants (Figure 2E). We further analysed hepatic expression of de novo lipogenesis fatty acid synthase (FAS) protein. Interestingly, chow‐fed Pla2g6^Hep−/−^ mice already showed a significant upregulation of FAS (Figure 2F). HFD feeding of Flox mice upregulated FAS expression which was not altered by myeloid‐ and hepatocyte‐PLA2G6 deficiency. Collectively, PLA2G6 deficiency in both cell types under basal conditions elicited an increase in plasma lipoproteins, plasma/liver LPS, liver lipids, plasma leptin and HOMA‐IR, all of which were exacerbated by HFD feeding despite reduced body and liver weights (Figure 1F).

### 3.3 Myeloid‐ and Hepatocyte‐PLA2G6 Deficiency Leads to Convergent Reduction of Macrovesicular Steatosis but Divergent Immune Activation Parameters in Blood After HFD Feeding

**FIGURE 3:**
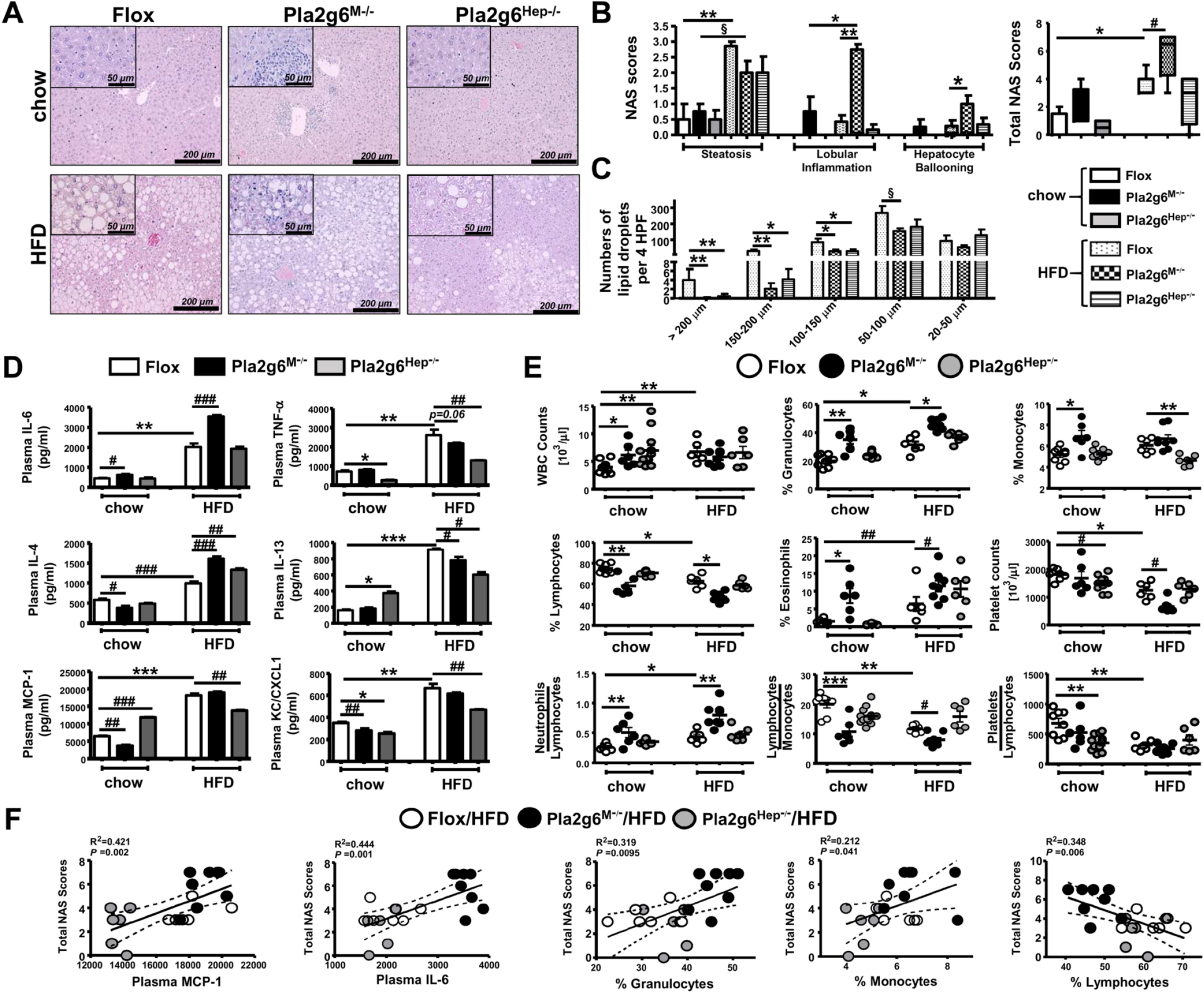
Pla2g6^M−/−^ and Pla2g6^Hep−/−^ mice under HFD similarly show attenuated hepatic macrovesicular lipid droplets but opposing phenotypes regarding liver inflammation, plasma cytokines and blood myeloid cells. Flox, Pla2g6^M−/−^ and Pla2g6^Hep−/−^ mice were fed with chow or HFD for 6 months. (A) Representative images of H&E‐stained livers (bar = 200 and 50 μm in insets), *N* ≥ 5 mice per group. (B) H&E‐stained livers were evaluated for steatosis, lobular inflammation, hepatocyte ballooning, and the total NAS combined. (C) The numbers of lipid droplets taken from 4 HPF‐pictures per slide/mouse were grouped according to diameter sizes of lipid droplets: > 200, 150–200, 100–150, 50–100 and 20–50 μm. (D) Plasma levels (pg/mL) of inflammatory cytokines IL‐6, TNF‐α, IL‐4, IL‐13, MCP‐1 and KC/CXCL1. (E) Blood WBC and platelet counts (10^3^/μL), the composition (%) of granulocytes, monocytes, lymphocytes and eosinophils as well as the ratios of neutrophils‐to‐lymphocytes, lymphocytes‐to‐monocytes and platelets‐to‐lymphocytes. (F) Correlation plots between total NAS scores and plasma MCP‐1, IL‐6, blood granulocytes, monocytes or lymphocytes. Data are mean ± SEM, *N* = 4–8 (B and C), *N* = 6–12 (D–F). *p < 0.05, **p < 0.01 and ***p < 0.001 by Kruskal–Wallis tests with Dunn's selected pair post‐tests; ^§^ *p* < 0.05 by one‐tailed or ^#^ *p* < 0.05, ^##^ *p* < 0.01, ^###^ *p* < 0.001 by two‐tailed Mann–Whitney *U* tests. H&E, haematoxylin & eosin staining; HFD, high‐fat diet; HPF, high‐power‐field; NAS, NAFLD activity scores; WBC, white blood cells.

Despite increased plasma lipoproteins in HFD‐fed iPLA2β‐null mice (Figure 2A), they showed an attenuation of liver TG and steatosis scores without affecting lobular inflammation [14]. Here, HFD‐fed Flox mice exhibited both macro‐ and microvesicular steatosis, and HFD‐fed Pla2g6^M−/−^ and Pla2g6^Hep−/−^ mice showed a trend toward decreased hepatic steatosis (Figure 3A,B, left panel). Notably, HFD‐fed Pla2g6^M−/−^ mice exhibited pronounced presence of mononuclear cell infiltrates and distinctive lymphoid aggregates (Figure 3A), accompanied by a further increase in lobular inflammation, hepatocyte ballooning and total NAFLD activity scores (NAS) (Figure 3B). These parameters were however not evident in HFD‐fed Pla2g6^Hep−/−^ mice. We further quantified the proportion of macro‐ and microvesicular steatosis by enumerating lipid droplets according to diameters. Under HFD, a significant reduction in the numbers of lipid droplets with large sizes of > 200, 150–200 and 100–150 μm was observed in both mutants (Figure 3C). Thus, myeloid‐ and hepatocyte‐PLA2G6 deficiency under HFD caused a reduction of large‐sized lipid droplets associated with increased lipoproteins (Figure 2B) and exacerbated liver TG (Figure 2D). Thus, the deficiency induced a shift in lipid transfer from lipid droplets to lipoprotein biogenesis since hepatocellular TG can be transferred between these two compartments.

Upon evaluation of blood cytokines, HFD‐fed Pla2g6^M−/−^ mice displayed a further increase in plasma IL‐6 and IL‐4, while HFD‐fed Pla2g6^Hep−/−^ mice displayed a significant attenuation of TNF‐α, IL‐13, MCP‐1 and keratinocyte chemoattractant/C‐X‐C motif chemokine receptor 1 (KC/CXCL1) (Figure 3D). These results indicated the divergent exacerbation and protection by myeloid‐ and hepatocyte‐PLA2G6 deficiency, respectively. Under chow, Pla2g6^M−/−^ mice displayed increased M1 IL‐6 but attenuated M2/Th2 IL‐4 and MCP‐1. In contrast, chow‐fed Pla2g6^Hep−/−^ mice displayed decreased TNF‐α but increased IL‐13 and MCP‐1. Both mutants under chow showed attenuation of KC/CXCL1. Upon evaluation of complete blood counts, HFD feeding of Flox mice increased the counts of white blood cells (WBC), granulocytes and eosinophils with decreased counts of lymphocytes and platelets (Figure 3E). Under HFD, Pla2g6^M−/−^ mice exhibited a further increase in granulocytes and eosinophils; however, Pla2g6^Hep−/−^ mice showed attenuation of monocytes. Under chow, both mutants showed an increase in WBC with Pla2g6^M−/−^ mice exhibiting a stark increase in granulocytes, monocytes and eosinophils. Pla2g6^M−/−^ mice under both diets exhibited increased neutrophils‐to‐lymphocytes ratio and attenuated lymphocytes‐to‐monocytes ratio. Chow‐fed Pla2g6^Hep−/−^ mice showed decreased platelet counts and platelets‐to‐lymphocytes ratio. A positive correlation was obtained between NAS and plasma MCP‐1, IL‐6, granulocytes or monocytes, as well as a negative correlation between NAS and blood lymphocytes (Figure 3F).

Collectively, despite reduced obesity in both mutants under HFD, Pla2g6^M−/−^ mice displayed exacerbation of inflammatory responses while Pla2g6^Hep−/−^ mice displayed protection against HFD‐induced changes in plasma cytokines/chemokines and WBC. Notwithstanding, the deficiency in these cell types under basal conditions induced an increase in cytokines/chemokines and WBC in a similar manner as metabolic parameters (Figure 2B–E).

### 3.4 Myeloid‐ and Hepatocyte‐PLA2G6 Deficiency Leads to an Opposing Response on Hepatic Programmed Cell Death and Inflammation After HFD Feeding

**FIGURE 4:**
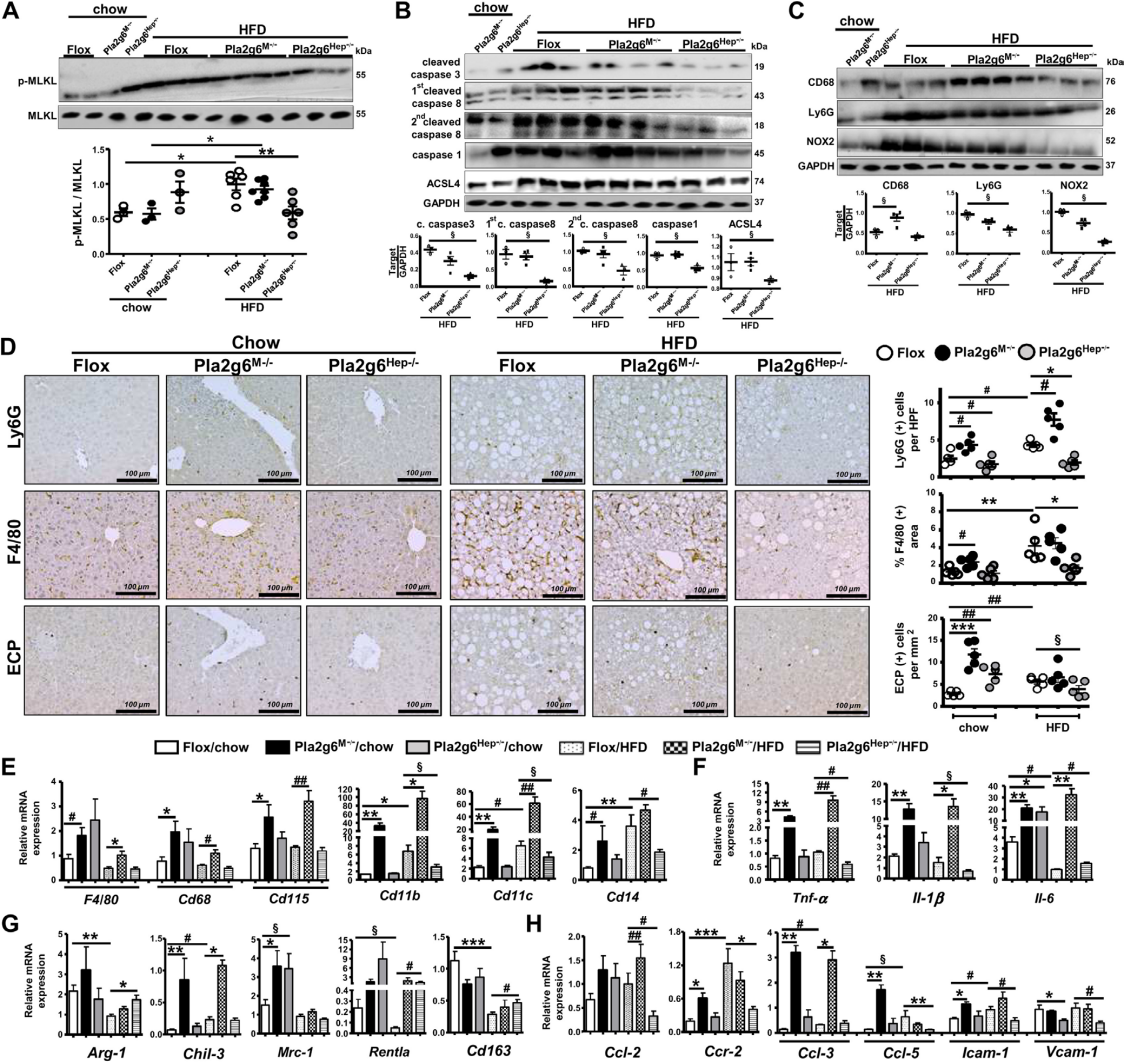
Pla2g6^M−/−^ and Pla2g6^Hep−/−^ mice under HFD show opposing phenotypes regarding hepatic programmed cell death and inflammatory response. Flox, Pla2g6^M−/−^ and Pla2g6^Hep−/−^ mice were fed with chow or HFD for 6 months. (A) Hepatic protein expression of p‐MLKL and loading control MLKL and quantification in mice fed with chow or HFD (*N* = 3–6). (B) Hepatic protein expression of cleaved caspase‐3, 1st cleaved caspase‐8 (43 kDa), 2nd cleaved caspase‐8 (18 kDa), caspase 1 and ACSL4 in mice under chow and HFD, and quantification in mice under HFD (*N* = 3–4). (C) Hepatic protein expression of CD68, Ly6G and NOX2 in mice under chow and HFD and quantification in mice under HFD (*N* = 3–4). (D) Representative images of hepatic Ly6G, F4/80 and ECP IHC‐staining (left), and quantification (right) in mice fed with chow or HFD (*N* = 5). RT‐qPCR analysis of hepatic (E) immune cells *F4/80, Cd68, Cd115, Cd11b, Cd11c* and *Cd14*. (F) M1 markers *Tnf‐α*, *Il‐1β* and *Il‐6*. (G) M2 markers *Arg‐1, Chil‐3, Mrc‐1, Rentla* and *Cd163*. (H) chemokines *Ccl‐2, Ccr‐2, Ccl‐3, Ccl‐5, Icam‐1* and *Vcam‐1* (*N* = 4–8). Data are mean ± SEM. *p < 0.05, **p < 0.01 and ***p < 0.001 by Kruskal–Wallis tests with Dunn's selected pair post‐tests; ^§^ *p* < 0.05 by one‐tailed or ^#^ *p* < 0.05, ^##^ *p* < 0.01 by two‐tailed Mann–Whitney *U* tests. ECP, eosinophil cationic protein; HFD, high‐fat diet; IHC, immunohistochemistry; RT‐qPCR, quantitative real‐time polymerase chain reaction.

We further determined the divergent phenotypes between Pla2g6^M−/−^ and Pla2g6^Hep−/−^ mice by considering hepatocellular programmed cell death, necroptosis, apoptosis, pyroptosis and ferroptosis. Under HFD, Pla2g6^Hep−/−^ but not Pla2g6^M−/−^ mice exhibited attenuated protein expression of phosphorylated mixed lineage kinase domain‐like protein (p‐MLKL), a terminal‐known obligate effector of necroptosis (Figure 4A). Compared to Flox counterparts, HFD‐fed Pla2g6^Hep−/−^ mice also displayed downregulated hepatic expression of apoptotic caspase‐3 and caspase‐8, pyroptosis marker caspase‐1 and ferroptosis marker acyl‐CoA synthetase long‐chain family member 4 (ACSL4) (Figure 4B) as well as lymphocyte antigen 6 family member G (Ly6G) and NADPH oxidase 2 (NOX2) (Figure 4C). In contrast, HFD‐fed Pla2g6^M−/−^ mice showed upregulation of macrophage marker CD68.

To further evaluate inflammatory events, we performed liver IHC staining in our mouse cohort. HFD feeding of Flox mice caused a significant increase in the positive staining of Ly6G, F4/80 and eosinophil cationic protein (ECP) as markers of neutrophils, macrophages and eosinophils, respectively (Figure 4D). Under HFD, Pla2g6^M−/−^ mice displayed a further increase in neutrophil infiltration, while Pla2g6^Hep−/−^ mice exhibited attenuation of these three immune cell types. Notably, Pla2g6^M−/−^ mice under chow already showed enhanced infiltration of these three immune cells (Figure 4D) concomitant with upregulation of immune cell markers *F4/80*, *Cd68*, *Cd115*, *Cd11b*, *Cd11c* and *Cd14* (Figure 4E), M1 *Tnf‐α*, *Il‐1β* and *Il‐6* (Figure 4F), M2 *Chil‐3* and *Mrc‐1* (Figure 4G) as well as chemokines (Figure 4H). Under HFD, Pla2g6^M−/−^ mice exhibited upregulation of all immune cell markers and M1 cytokines (Figure 4E,F) as well as *Chil‐3*, *Ccl‐2* and *Ccl‐3* (Figure 4G,H). While chow‐fed Pla2g6^Hep−/−^ mice showed upregulation of *Il‐6* (Figure 4F), these mutants under HFD exhibited downregulation of *Cd11b*, *Cd11c* and *Cd14* (Figure 4E), *Tnf‐α* and *Il‐1β* (Figure 4F), chemokines (Figure 4H) concomitant with upregulation of M2 markers (Figure 4G). Thus, the exacerbation of HFD‐induced hepatic inflammation in Pla2g6^M−/−^ mice was in line with the increase in metabolic parameters (Figure 2B–E). However, HFD‐fed Pla2g6^Hep−/−^ mice displayed dissociation between the metabolic parameters and hepatocellular programmed cell death and inflammation.

### 3.5 Myeloid‐ and Hepatocyte‐PLA2G6 Deficiency Modulates Hepatic Lymphopoiesis in an Opposite Manner After HFD Feeding

**FIGURE 5:**
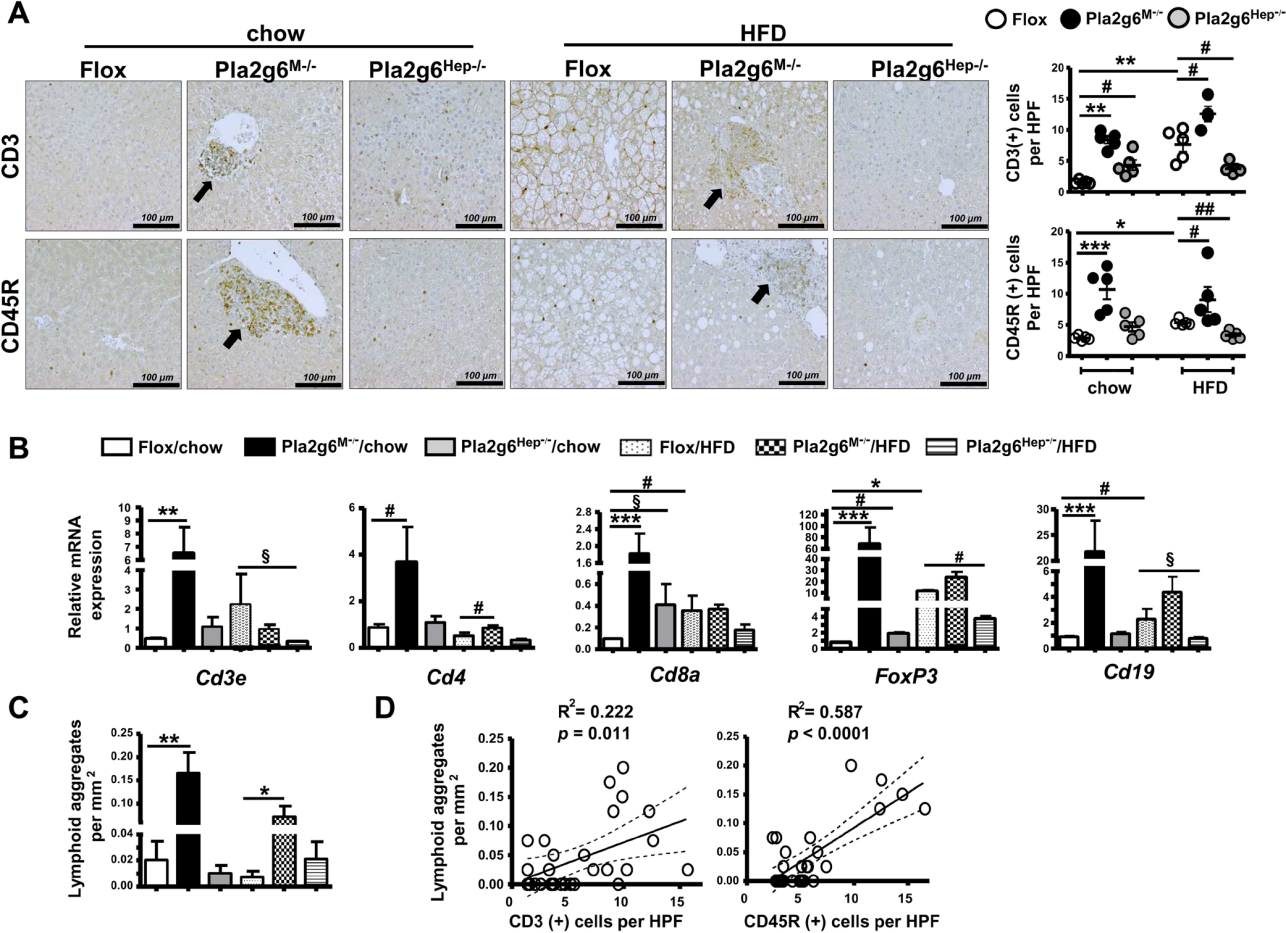
Pla2g6^M−/−^ and Pla2g6^Hep−/−^ mice under HFD show opposing hepatic lymphopoietic response. Flox, Pla2g6^M−/−^ and Pla2g6^Hep−/−^ mice were fed with chow or HFD for 6 months. (A) Representative images of CD3 and CD45R IHC‐staining (left) and quantification (right) in mice fed with chow or HFD. Black arrows indicate infiltrated lymphoid aggregates. (B) RT‐qPCR analysis of hepatic T and B cells *Cd3e, Cd4, Cd8a, FoxP3* and *Cd19*. (C) The numbers of lymphoid aggregates per liver mm^2^. (D) Correlation plots showing correlation coefficients *R* ^2^ and *p* values between the numbers of lymphoid aggregates per mm^2^ and CD3 (+) or CD45R (+) cells per HPF. Data are mean ± SEM, *N* = 4–5 (A and D) and *N* = 4–8 (B and C). *p < 0.05, **p < 0.01 and ***p < 0.001 by Kruskal–Wallis tests with Dunn's selected pair post‐tests; ^§^ *p* < 0.05 by one‐tailed or ^#^ *p* < 0.05, ^##^ *p* < 0.01 by two‐tailed Mann–Whitney *U* tests. HFD, high‐fat diet; HPF, high‐power‐field; RT‐qPCR, quantitative real‐time polymerase chain reaction.

We previously demonstrated that male Pla2g6^M−/−^ mice under basal and LPS treatment displayed hepatic lymphoplasmacellular infiltration with IHC positivity of CD3 and CD45R for T and B cells, respectively [20]. Here we showed that HFD feeding of Flox mice increased hepatic positivity of T and B lymphopoiesis, which was further increased in Pla2g6^M−/−^ but attenuated in Pla2g6^Hep−/−^ mice (Figure 5A). The latter was confirmed by downregulated mRNA expression of *Cd3e*, *FoxP3* and *Cd19* as markers of T cells, regulatory T cells and B cells, respectively (Figure 5B). Notably, chow‐fed Pla2g6^M−/−^ mice exhibited CD3 and CD45R positivity (Figure 5A) as well as an upregulation of *Cd3e*, T helper *Cd4*, cytotoxic T cell *Cd8a*, *FoxP3* and *Cd19* (Figure 5B).

Beyond its metabolic functions, the liver maintains a distinct immune homeostasis characterized by specialized lymphoid aggregates consisting of ectopic lymphoid‐like structures and intrahepatic myeloid aggregates for T‐cell expansion [26]. In our study, Pla2g6^M−/−^ but not Pla2g6^Hep−/−^ mice under both chow and HFD exhibited these distinctive lymphoid aggregates (Figure 5C). These structures were predominantly localized in perivascular and periportal regions and displayed a poorly organized architecture (black arrows, Figure 5A). A strong positive linear correlation was observed between the numbers of hepatic lymphoid aggregates and the positivity of CD3 or CD45R (Figure 5D), indicating that hepatic T and B cells were constituted into the lymphoid aggregates. Thus, together with hepatic M1/innate inflammation (Figure 4C–H), Pla2g6^M−/−^ mice under basal conditions and HFD feeding displayed aberrant hepatic lymphopoiesis, and on the contrary, Pla2g6^Hep−/−^ mice displayed protection against HFD‐induced hepatic lymphopoiesis.

### 3.6 Myeloid‐ and Hepatocyte‐PLA2G6 Deficiency Regulates Hepatic Fibrosis in an Opposite Manner After HFD Feeding

**FIGURE 6:**
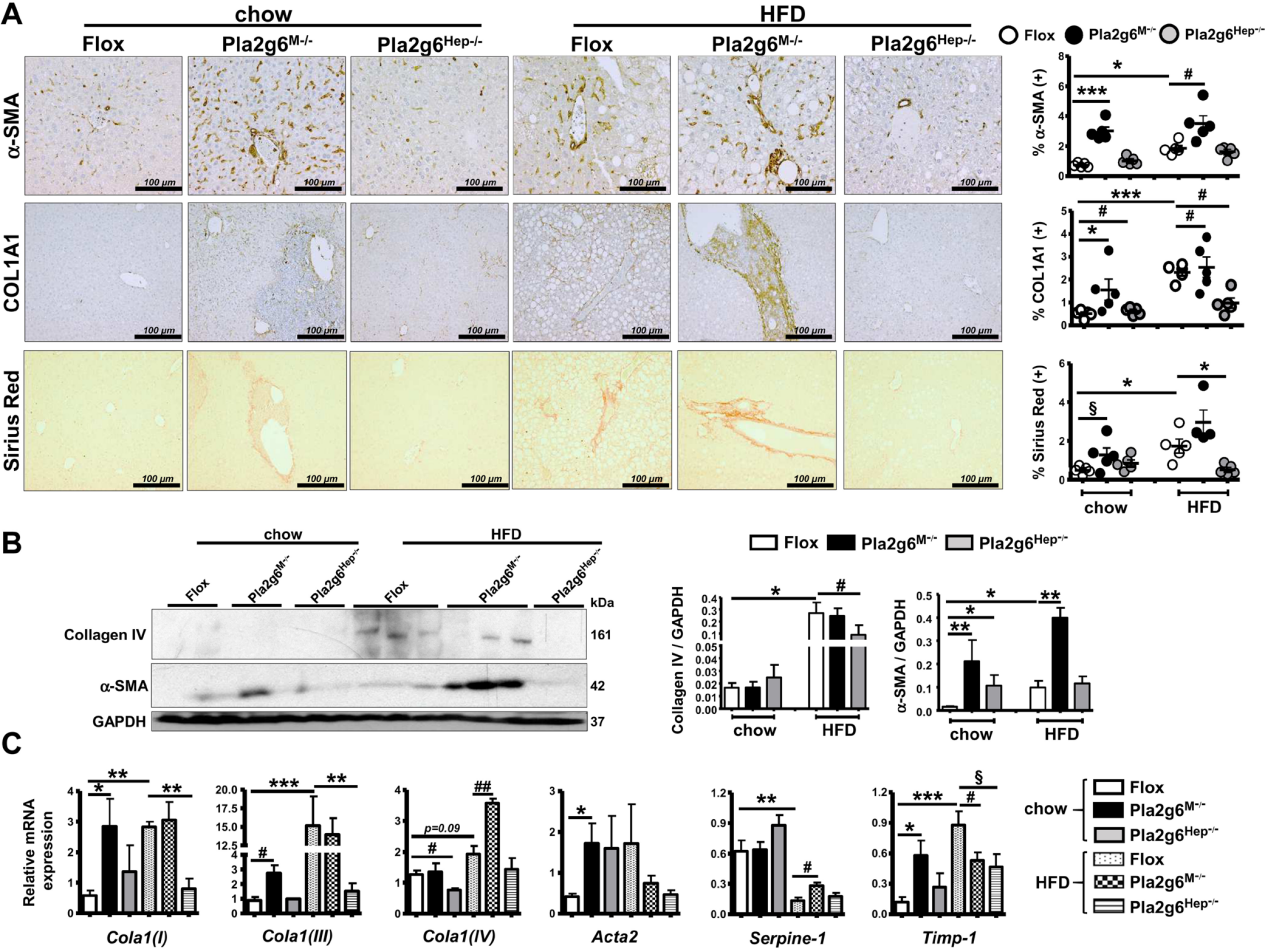
Pla2g6^M−/−^ and Pla2g6^Hep−/−^ mice fed HFD show opposing hepatic fibrosis response. Flox, Pla2g6^M−/−^ and Pla2g6^Hep−/−^ mice were fed with chow or HFD for 6 months. (A) Representative images of α‐SMA, COL1A1 and Sirius‐Red staining (left) and quantification (right, *N* = 5). (B) Western blot analysis of hepatic protein expression (left) and quantification (right) of collagen IV and α‐SMA. (C) RT‐qPCR analysis of hepatic fibrosis genes *Cola1(I)*, *Cola1(III)*, *Cola1(IV)*, *Acta2, Serpine‐1* and *Timp1*. Data are mean ± SEM, *N* = 3–8 (B and C). *p < 0.05, **p < 0.01 and ***p < 0.001 by Kruskal–Wallis tests with Dunn's selected pair post‐tests; ^§^ *p* < 0.05 by one‐tailed or ^#^ *p* < 0.05, ^##^ *p* < 0.01 by two‐tailed Mann–Whitney *U* tests. COL1A1, collagen IA1; HFD, high‐fat diet; RT‐qPCR, quantitative real‐time polymerase chain reaction.

In nonobese MASH model without insulin resistance generated by MCD diet [19], we previously demonstrated an exacerbation and protection against hepatic fibrosis in female Pla2g6^M−/−^ and Pla2g6^Hep−/−^ mice, respectively [18]. In our current cohort, HFD feeding of Flox mice increased fibrotic positivity of hepatic α‐SMA and collagen1A1 (COL1A1) as well as Sirius‐Red staining (Figure 6A). Under HFD, Pla2g6^Hep−/−^ mice exhibited a marked decrease in COL1A1 and Sirius‐Red staining, while Pla2g6^M−/−^ mice displayed a further increase in α‐SMA and COL1A1. Under HFD, Pla2g6^Hep−/−^ mice displayed downregulation of collagen IV protein, while Pla2g6^M−/−^ mice displayed further upregulation of α‐SMA protein (Figure 6B). On the mRNA level, HFD feeding of Flox mice induced upregulation of hepatic *Cola1(I)*, *Cola1(III)* and *Timp‐1* expression, which was attenuated in Pla2g6^Hep−/−^ mice (Figure 6C). On the contrary, HFD‐fed Pla2g6^M−/−^ mice exhibited further upregulation of *Cola1(IV)* and *Serpine1*. In line with the results in Figure 6A,B, Pla2g6^M−/−^ mice under chow displayed upregulation of fibrosis genes. Taken together, myeloid‐PLA2G6 deficiency promoted HFD‐induced hepatic fibrosis, while the deficiency in hepatocytes elicited protection. These data were consistent with hepatic inflammation and lymphopoiesis (Figures 4 and 5).

### 3.7 Hepatocyte‐PLA2G6 Deficiency Leads to Metabolic Alterations of Hepatic TG/Lipoprotein Synthesis and *de novo* Lipogenesis After HFD Feeding

**FIGURE 7:**
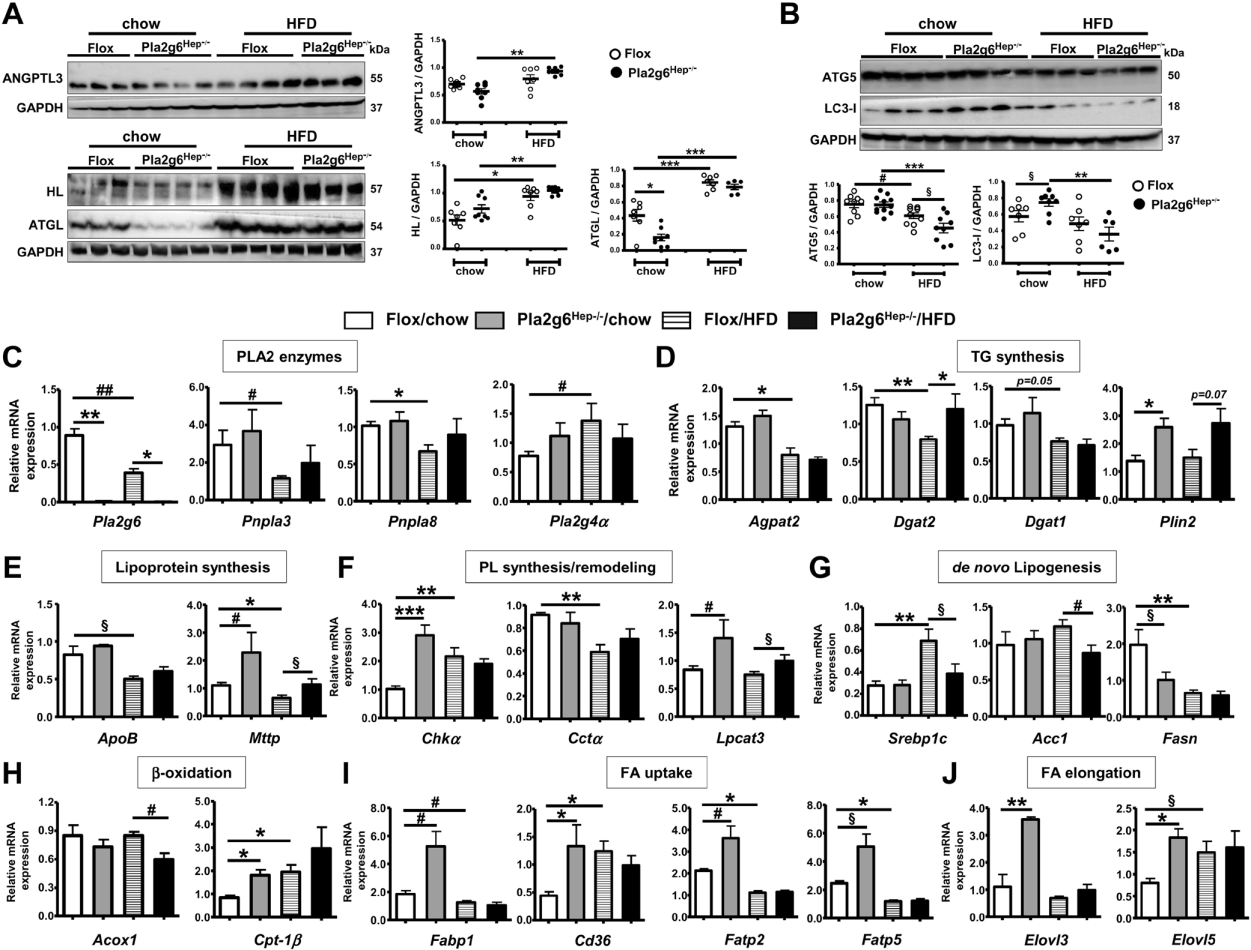
Pla2g6^Hep−/−^ mice under HFD show a rescue of the loss of hepatic autophagy and TG/lipoprotein synthesis genes concomitant with attenuation of de novo lipogenesis genes. In this study, Flox and Pla2g6^Hep−/−^ mice fed with chow or HFD were analysed. (A) Hepatic protein expression of an inhibitor of lipoprotein lipases (ANGPTL3) and TG/lipoprotein lipase (ATGL and HL) as well as quantification (*N* = 6–8). (B) Hepatic protein expression of autophagy (ATG5 and LC3‐I) and quantification (*N* = 6–8). Hepatic mRNA expression of (C) PLA2 genes *Pla2g6, Pnpla3, Pnpla8* and *Pla2g4α*. (D) TG synthesis genes *Agpat2, Dgat2, Dgat1* and *Plin2*. (E) lipoprotein assembly and secretion genes *ApoB* and *Mttp*. (F) PL synthesis genes *Chkα*, *Cctα* and *Lpcat3*. (G) de novo lipogenesis genes *Srebp1c, Acc1* and *Fasn*. (H) β‐oxidation genes *Acox1* and *Cpt‐1β*. (I) FA uptake genes *Fabp1, Cd36, Fatp2* and *Fatp5*. (J) FA elongase genes *Elovl3* and *Elovl5* (*N* = 4–7). Data are mean ± SEM. *p < 0.05, **p < 0.01 and ***p < 0.001 by Kruskal–Wallis tests with Dunn's selected pair post‐tests; ^§^ *p* < 0.05 by one‐tailed or ^#^ *p* < 0.05, ^##^ *p* < 0.01 by two‐tailed Mann–Whitney *U* tests. FA, fatty acids; HFD, high‐fat diet; PL, phospholipids; PLA2, phospholipase A2; TG, triglycerides.

To this end, Pla2g6^Hep−/−^ mice under basal conditions showed increased plasma lipoproteins/LPS, liver LPS/lipids and HOMA‐IR (Figure 2), which were further increased in these mutants under HFD despite remarkable protection against hepatic inflammatory fibrosis (Figures 3, 4, 5, 6). We therefore investigated metabolic pathways to delineate specific phenotypes of Pla2g6^Hep−/−^ mice under chow and HFD. With elevation of plasma lipoproteins (Figure 2B), HFD‐fed Pla2g6^Hep−/−^ mice showed a decrease in steady‐state plasma TG, Chol and NEFA (Figure 1I), suggesting accelerated lipoprotein clearance, a process catalysed by lipoprotein lipases. Upon western blot analysis of an inhibitor of lipoprotein lipases, angiopoietin‐like 3 (ANGPTL3), HFD‐fed Pla2g6^Hep−/−^ mice, however, did not show any alteration of this protein (Figure 7A). Furthermore, HFD feeding of Flox mice induced marked upregulation of hepatic lipase (HL) and ATGL capable of hydrolyzing TG in plasma lipoproteins and lipid droplets, respectively. Hepatic expression of these two proteins was also not disturbed in Pla2g6^Hep−/−^ mice under HFD (Figure 7A). Thus, the reduction of plasma TG and NEFA in HFD‐fed Pla2g6^Hep−/−^ mice did not involve lipoprotein clearance nor TG hydrolysis in lipid droplets, but was likely due to inherent protection against HFD‐induced hepatic programmed cell death, inflammation and fibrosis (Figures 4, 5, 6).

It is known that autophagy regulates catabolism of hepatic lipid droplets and lipoproteins, and its suppression drives hyperinsulinemia and insulin resistance [27]. Upon analysis of autophagic proteins, HFD‐fed Flox mice exhibited downregulation of autophagy‐related gene 5 (ATG5), which was further downregulated in Pla2g6^Hep−/−^ mice (Figure 7B). Pla2g6^Hep−/−^ mice under chow, however, showed moderate upregulation of microtubule‐associated protein light chain 3‐I (LC3‐I), which was significantly attenuated upon HFD feeding of this mutant. Thus, Pla2g6^Hep−/−^ mice under HFD exhibited further impairment of autophagy, which was in line with exacerbation in HOMA‐IR (Figure 2E), highlighting the involvement of autophagy in lipoprotein biogenesis and insulin resistance.

We further analysed hepatic mRNA expression of genes related to metabolism. Associated with upregulated expression of iPLA2β/PLA2G6 protein (Figure 1A), HFD feeding of Flox mice led to downregulation of *Pla2g6*, *Pnpla3* and group VIB PLA2 (*Pnpla8*) concomitant with upregulation of cPLA2α (*Pla2g4a*) (Figure 7C). These genes were however not affected by hepatocyte‐PLA2G6 deficiency indicating an absence of compensatory mechanisms by these PLA2s. Via feedback regulation, HFD feeding of Flox mice induced downregulation of enzymes involved in TG biosynthesis *Agpat2*, *Dgat1* and *Dgat2* (Figure 7D) as well as genes related to VLDL assembly and secretion *ApoB* and *Mttp* (Figure 7E). Interestingly, significant upregulation of perilipin2 (*Plin2*) and *Mttp* was observed in Pla2g6^Hep−/−^ mice under chow and HFD (Figure 7D,E), thus linking hepatocellular PLA2G6 with lipid‐droplet dynamics and lipoprotein biogenesis.

Compared to Flox counterparts, PL synthesis genes *Chkα* and *Cctα* were unchanged in HFD‐fed Pla2g6^Hep−/−^ mice (Figure 7F), whereas de novo lipogenesis genes *Srebp1c* and *Acc1* were downregulated (Figure 7G). This downregulation was also observed in Pla2g6^Hep−/−^ mice fed MCD diet [18]. Similar to *Plin2* and *Mttp*, Pla2g6^Hep−/−^ mice under chow showed a significant upregulation of *Chkα* and *Lpcat3* (Figure 7F), and the latter gene was also upregulated in these mutants under HFD. Remarkably, Pla2g6^Hep−/−^ mice under chow also displayed significant upregulation of genes related to β‐oxidation (Figure 7H), FA uptake (Figure 7I) and elongation (Figure 7J), whereas these genes were not altered in these mutants under HFD. Thus, hepatocyte‐PLA2G6 deficiency under normal conditions activated hepatic lipoprotein biogenesis, PL synthesis and remodelling as well as FA uptake and elongation, and these events were correlated with a moderate increase in AST and plasma lipids (Figure 1), plasma lipoproteins, plasma/liver LPS, liver lipids and HOMA‐IR (Figure 2). While detrimental under normal conditions, hepatocyte‐PLA2G6 deficiency however protected against HFD‐induced hepatic programmed cell death, lymphopoiesis and inflammatory fibrosis (Figures 3, 4, 5, 6).

## 4 Discussion

We demonstrated that PLA2G6 deficiency in myeloid cells and hepatocytes led to opposing outcomes which contributed to the mixed phenotypes in iPLA2β‐null mice under HFD [14]. Pla2g6^M−/−^ mice displayed significant hepatic recruitment of granulocytes and lymphocytes in a stepwise manner from chow to HFD feeding. In the latter case, Pla2g6^M−/−^ mice showed an increase in plasma cytokines, plasma lipids, liver inflammation and fibrosis, as well as, metabolic parameters, that is, plasma lipoproteins, plasma/liver LPS, liver lipids, plasma leptin/insulin and HOMA‐IR. While Pla2g6^Hep−/−^ mice displayed an increase in these metabolic parameters in a stepwise manner from chow to HFD, however, they were protected against HFD‐induced hepatic programmed cell death and inflammatory fibrosis. Thus, PLA2G6 inactivation in hepatocytes appears to be an attractive strategy for targeted treatment of MASLD/MASH patients particularly those with inflammation‐driven and fibrosis‐prone disease [28].

It is known that cytokine secretion by macrophages requires synthesis of PL indicating that modulation of PL metabolism [29] and the release of AA [16] can regulate inflammatory response. Consistently, PL accumulation in BMDM from Pla2g6^M−/−^ mice led to an increased release of TNF‐α, and that in vivo LPS treatment of Pla2g6^M−/−^ mice primed their BMDM to exhibit a shift from TNF‐α toward chemokine MIP‐1α. The shift from M1 to M2/chemokine MCP‐1 was also observed in LPS + IFNγ‐treated macrophages from iPLA2β‐null mice [17]. Furthermore, PL accumulation in BMDM from Pla2g6^M−/−^ mice can also lead to an increase in eicosanoid PGE2 and lipoxin A4 via AA‐PL hydrolysis by cPLA2α [30]. BMDM activation of chemokines and eicosanoids [18, 30] may promote hepatic recruitment of phagocytes observed in Pla2g6^M−/−^ mice in our study. During metabolic endotoxemia [23], these cytokines/chemokines released by Pla2g6^M−/−^ macrophages may in turn stimulate de novo lipogenesis in liver [31], the release of insulin by pancreatic beta‐cells [32], and the release of leptin by adipocytes [33]. Metabolic LPS may also promote the secretion of lipoproteins by hepatocytes [34], and these lipoproteins may subsequently increase insulin secretion by pancreatic beta‐cells [35]. As Pla2g6^M−/−^ mice were sensitive to LPS [20], this series of events initiated by cytokines could result in an increase in all metabolic parameters observed in Pla2g6^M−/−^ mice in a stepwise manner from chow to HFD (Figure 2B–E). Unlike male Pla2g6^M−/−^ mice in the current study, female Pla2g6^M−/−^ mice under either chow or MCD diet however did not show any metabolic changes in plasma and liver TG [18], likely owing to the protective effects of oestrogen against MASLD. Herein, male Pla2g6^M−/−^ mice also displayed marked infiltration of neutrophils, macrophages, T and B lymphocytes in a stepwise manner from chow to HFD, and without disturbing programmed cell death and apoptosis under HFD. Hepatic recruitment of these immune cells is likely due to signalling from MIP‐1α and other chemokines during metabolic endotoxemia, which may activate hepatic stellate cells and exacerbate liver fibrosis [20]. Thus, myeloid‐PLA2G6 deficiency provided the microenvironments of cytokines and chemokines that may underlie MASH with inflammatory fibrosis and whole‐body insulin resistance.

While chow‐fed iPLA2β‐null mice showed no alterations in liver PL [14], chow‐fed Pla2g6^Hep−/−^ mice displayed an increase in hepatic PL, particularly PE and PI containing AA, which led to an increase in lipoxin A4 following cPLA2α‐catalysed hydrolysis of AA‐PL. It is known that the biosynthesis and secretion of lipoproteins is regulated by hepatocellular PC [36] and PS [37]. Consistently, the increased PC and PS in Pla2g6^Hep−/−^ hepatocytes promoted a stepwise elevation of TG‐rich VLDL/LDL and VLDL/LDL/HDL‐Chol from chow to HFD, and the latter was in line with HFD‐fed iPLA2β‐null mice with exon 2 deletion [21]. It has been shown that a third of lipoprotein‐associated PC is converted into hepatic TG [25], thus an increase in hepatic PC and PS and secreted lipoproteins in Pla2g6^Hep−/−^ mice could significantly contribute to an increase in liver TG in a stepwise manner from chow to HFD. Lipoproteins are able to stimulate insulin secretion by pancreatic beta‐cells [35] and insulin is the major regulator of leptin production by adipocytes. Similar to lipoproteins, plasma leptin was elevated in Pla2g6^Hep−/−^ mice in a stepwise manner from chow to HFD. Because leptin is also a key regulator of body adiposity, the elevation of leptin observed in HFD‐fed Pla2g6^M−/−^ and Pla2g6^Hep−/−^ mice was correlated with reduction of body and liver weights as well as large‐sized lipid droplets in their livers. The mechanism for the latter case may involve an arrest of lipid droplets in the ER membrane by tight binding of lipidated ApoB to its luminal surface [38]. Notably, Pla2g6^Hep−/−^ mice under basal conditions displayed marked upregulation of FAS protein and mRNA of genes related to lipid droplets, FA uptake and FA elongation that led to lipotoxicity, as reflected by elevated AST. The mechanisms for such upregulation may involve PE‐dependent lipid droplet biosynthesis [39] and hepatic transcriptional activation of FA uptake and elongation genes induced by lipotoxicity [40]. Collectively, the increase in lipoproteins in Pla2g6^Hep−/−^ mice with a progressive pattern from chow to HFD feeding was critical for the development of hyperleptinemia and hyperinsulinemia.

Despite hyperleptinemia and insulin resistance, HFD‐fed Pla2g6^Hep−/−^ mice were protected against hepatocellular cell death and inflammatory fibrosis with attenuation of plasma lipids and cytokines as well as hepatic elevation of cytoprotective lipoxin A4. The latter was also observed in Pla2g6^Hep−/−^ mice under MCD diet [18]. PANoptosis is a coordinated cell death pathway that involves pyroptosis, apoptosis and necroptosis, and this pathway is also linked to ferroptosis [41]. HFD‐fed Pla2g6^Hep−/−^ mice displayed attenuation of all components of PANoptosis and ferroptosis with remarkable attenuation of phosphorylated MLKL but not total MLKL protein. As previously hypothesized [14], hepatocyte‐PLA2G6 deficiency may replenish the loss of hepatic PL during MASLD/MASH, and this replenishment may support the repair at plasma membrane, the site where phosphorylated MLKL initiates cell death. The repair system removes phosphorylated MLKL in plasma membrane through endocytosis and exocytosis mediated by endosomal sorting complex required for transport (ESCRT) [42]. More relevantly, ESCRT association with the membrane is mediated by electrostatic interactions with anionic PL, such as PS, to facilitate the high membrane curvature, invagination and scission in initiating exocytosis [43]. ESCRT‐dependent membrane repair is able to block pyroptosis and ferroptosis thus linking these pathways with necroptotic MLKL activation [44]. On the contrary, Pla2g6^Hep−/−^ mice under chow exhibited supraphysiological accumulation of PL and rendered PL containing polyunsaturated fatty acids susceptible to peroxidation [45] potentially explaining the elevated phosphorylated MLKL, caspase 1, and AST in these mice. Further investigations are needed to demonstrate ESCRT‐dependent repair of damaged membrane that could result in PANoptosis protection in HFD‐fed Pla2g6^Hep−/−^ mice.

Our male Pla2g6^M−/−^ and Pla2g6^Hep−/−^ mice under HFD manifested as nondiabetic nonobese MASLD because they displayed reduced blood glucose and body/liver weights, but elevated plasma lipoproteins, hyperleptinemia and hyperinsulinemia. Emerging data support a link between MASLD and neurodegeneration [46] through liver‐brain axis [47]. Hence, the observed elevation of plasma lipids and leptin/insulin resistance in both mutants under HFD may contribute to neuroinflammation by global PLA2G6 inactivation seen in iPLA2β‐null mice [9] and patients with PLA2G6 mutations [11]. The overall genetic prevalence of PLA2G6‐associated neurodegeneration is estimated to be 1 in 1 million with the highest prevalence among African/African‐Americans and East‐Asians [48]. Our results may be applicable to these patients who consume excessive dietary fat and exhibit MASLD/MASH without obesity. Moreover, our results also highlight the notion that the opposing functions across different cell types by global PLA2G6 inactivation could counterbalance potential protective mechanisms in specific cell populations. Hence, systemic use of iPLA2β/PLA2G6 inhibitors may not produce complete protection against MASH due to the detrimental effects from PLA2G6 inactivation in myeloid cells.

iPLA2β‐specific inhibitors such as FKGK18 have been developed, and FKGK18 is shown to treat autoimmune Type‐1 diabetes in NOD female mice [49]. Our work suggests that selective inactivation of PLA2G6 in hepatocytes may potentially provide a more precise therapeutic strategy for targeted treatment of patients with MASLD/MASH who are inflammation‐driven and fibrosis‐dominant [28]. To this end, suppression of liver‐specific PLA2G6 utilizing the short‐hairpin RNA knockdown and adenovirus‐vector technique has already been shown to protect mice from HFD‐induced MASLD [50].

## 5 Conclusion

Myeloid‐PLA2G6 deficiency induced hepatic recruitment of immune cells whereas the deficiency in hepatocytes led to mild metabolic abnormalities under basal conditions. After HFD feeding, myeloid‐PLA2G6 deficiency exacerbated hepatic inflammation and fibrosis while the deficiency in hepatocytes attenuated these events. PLA2G6 deficiency in both cell types, however, induced hyperleptinemia and hyperinsulinemia. Our findings underscore cell‐specific PLA2G6 targeting, and PLA2G6 inactivation in hepatocytes may hold a promising therapeutic strategy for treatment of MASH.

## Author Contributions

G.L.: data curation, formal analysis, investigation, methodology, writing – original draft. S.S.: formal analysis, investigation, methodology, writing – review and editing. S.T.‐K.: formal analysis, methodology, writing – review and editing. U.M.: conceptualization, investigation, software, supervision, writing – review and editing. W.C.: conceptualization, formal analysis, funding acquisition, supervision, writing – review and editing.

## Funding

This study was supported by Wuhan‐Heidelberg Heinz Götze Memorial Fellowship scholarships to G.L. WC acknowledges the funding from Deutsche Forschungsgemeinschaft (CH 288/6‐2) for the generation of tissue‐specific Pla2g6‐deficient mice. The funding sources had no involvement in study design; in the collection, analysis, interpretation of data; in writing of the report; and in the decision to submit the manuscript for publication.

## Ethics Statement

Studies involving animals were approved by the University of Heidelberg Institutional Animal Care and Use Committee, licence number 35–9185.81/G208/19.

## Conflicts of Interest

The authors declare no conflicts of interest.

## Supporting information


**Appendix S1:** Materials and methods.

## Data Availability

Analysed data on lipidomic profiling can be found at https://doi.org/10.6084/m9.figshare.31281208. The data that support the findings of this study are available on request from the corresponding author.
