# Supplementary material for: Distinct Roles of Myeloid‐ and Hepatocyte‐PLA2G6 Deletion in Mice With Metabolic Dysfunction‐Associated Steatotic Liver Disease
Source: Liver Int. 2026 May 8;46:e70679. doi: 10.1111/liv.70679 (PMC13155075; doi:10.1111/liv.70679)
Supplement: Supplementary file 1 — Appendix S1: Materials and methods. [file LIV-46-0-s001.docx]

Distinct Roles of Myeloid- and Hepatocyte-PLA2G6 deletion in Mice with Metabolic Dysfunction-associated Steatotic Liver Disease

Gang Li, Simone Staffer, Sabine Tuma-Kellner, Uta Merle, Walee Chamulitrat

**Appendix S1:**

**Material and Methods**

**Animals and HFD Feeding**

iPla2β-null mice with deletion of exon 9 were kind gifts from Dr. John Turk (Washington University School of Medicine, MS, USA). C57BL/6 (as WT controls) and iPla2β-null mice were bred at the animal facility of the University Heidelberg and genotyped according to our previous study [1]. Cross-breeding with C57BL/6 background of more than 20 back-crossings was performed prior to use. For generation of conditional knockout mice, Pla2g6^flox^ mice were first generated by Gen-O-Way S.A. (Lyon Cedex, France) as previously described [2]. Pla2g6^flox/flox^ mice were produced following an embryo transfer of heterozygous Pla2g6^flox^ mice. Upon Cre/loxP-mediated recombination, Pla2g6^flox/flox^ mice were cross-bred with *LysM-Cre* and *Alb-Cre* lines resulting in Pla2g6^Δex6–8^alleles to generate Pla2g6^M-/-^ and Pla2g6^Hep-/-^ mice, respectively. The generation of Pla2g6^flox/flox^ was approved by the University Heidelberg Animal Care and Use Committee and the German Authority (Baden-Württemberg Regierungspräsidium Karlsruhe) with license number 35-9185.81/G39/14. All these mouse lines were housed in a transgenic unit in the Interfakultäre Biomedizinische Forschungseinrichtung of the University of Heidelberg.

Male WT and iPla2β-null mice (6-12 mice per group) [22] as well as male Pla2g6^flox/flox^ (as Flox controls), Pla2g6^M-/-^, and Pla2g6^Hep-/-^ mice (6-13 mice per group) of 6 months old were fed with HFD diet (D12492 containing 34.9% w/w fat, Research Diet, NJ, USA) or chow (LASQC diet Rod18 containing 5% w/w fat, LASvendi, Soest, Germany) for 24 weeks. Cohorts in current study were consisted of Flox/chow (N = 8), Pla2g6^M-/-^/chow (N = 6), Pla2g6^Hep-/-^/chow (N = 13), Flox/HFD (N = 7), Pla2g6^M-/-^/HFD (N = 8), and Pla2g6^Hep-/-^/HFD (N = 6). After 24 weeks of feeding, body weights of euthanized mice were obtained following 4-hour fasting. Blood was collected for determination of complete-blood-cells counts, and plasma samples were prepared from vena cava blood and were kept in -80°C. The weights of liver, spleen, subcutaneous (inguinal) fat, and visceral (retroperitoneal and perirenal) fat were obtained. Liver samples were either fixed in 10% formalin or snap-frozen and stored at -80°C. In another cohort, male Flox and Pla2g6^M-/-^mice were intraperitoneally injected with saline or 1 mg/kg *E. coli* LPS (O111:B4, Sigma-Aldrich, Taufkirchen, Germany) for 24 h. According to the Animal Welfare Laboratory Animal Ordinance from the German Animal Welfare Act, HFD-feeding experiments were approved by the Animal Care and Use Committee of the University of Heidelberg and the German Authority (Baden-Württemberg Regierungspräsidium Karlsruhe) with license number 35-9185.81/G248/11 (for WT and iPla2β-null mice) and 35-9185.81/G208/19 (for Flox, Pla2g6^M-/-^, and Pla2g6^Hep-/-^ mice).

**Preparation of BMDM**

BMDM were prepared from bone marrow cells isolated from femurs and tibias of Flox and Pla2g6^M-/-^ mice treated with saline or 1 mg/kg LPS for 24 h, according to our previous studies [3]. Briefly, bone-marrow cells were subjected to red blood cell lysis and cultured overnight in complete RPMI-1640 containing penicillin/streptomycin and 10% FBS, supplemented with 20 ng/ml recombinant murine macrophage colony-stimulating factor (M-CSF, PeproTech) for 4 h. The adhered stromal cells and mature resident bone marrow macrophages were discarded. The floating bone-marrow cells were collected and differentiated to BMDM by culturing undisturbed in complete RPMI-1640 containing antibiotics, 3.3% FBS, and 20 ng/ml M-CSF for 3 days. On day 4 to day 7, BMDM were cultured in serum-free RPMI-1640 containing antibiotics, 10% pyruvate, and 20 ng/ml M-CSF. BMDM were trypsinized and plated for experiments.

**LC/MS-MS Profiling of PL**

Lipids from BMDM and liver samples were extracted by using hexane:isopropanol (3:2 v/v) containing internal standards. The profiles of 164 PL and SM were obtained by liquid-chromatography mass spectrometry (LC-MS/MS) with running conditions described previously [4]. Internal standards were phosphatidylcholine (PC)-17:0/17:0, phosphatidylethanolamine (PE)-12:0/12:0, phosphatidylserine (PS)-17:0/17:0, phosphatidylinositol (PI)-17:0/17:0, and ceramide-17:0 (Avanti Polar Lipids, Alabaster, AL, USA). Internal standard peak areas were monitored for quality control and used for quantification of analytes of samples and standards. Data acquisition and processing were performed with Masslynx version 4.1 software. The data were exported to Excel sheets and analyte/internal standard ratios were used to determine the response normalized to cellular mg protein for BMDM or nmol/mg for liver samples.

**Blood and Lipid Assays**

After sacrifice, EDTA-treated blood was subjected to determination of complete-blood-cells counts using a Scil Vet abc Plus+ hematology analyzer (Scil animal care company GmbH, Viernheim, Germany). Blood glucose levels were measured using an Accu-Chek Aviva (Roche, Mannheim, Germany). Plasma activities of alanine aminotransferase (ALT) and aspartate aminotransferase (AST) were determined using Randox kits (Krefeld, Germany). Homogenates of liver were subjected to Folch lipid extraction. After drying, lipid extracts were dissolved in chloroform containing 1% Triton X-100 which were again dried and reconstituted in deionized water which was used for lipid analyses [5]. Plasma samples or lipid extracts were subjected to the analyses of TG and NEFA using LabASSAY™ TG and NEFA-HR kits (Wako Chemicals GmbH, Neuss, Germany), respectively. Plasma total Chol levels were determined with a commercial kit from Randox (Krefeld, Germany). Lipoprotein profiles of iPLA2β-null mice under chow or HFD were analyzed by using an on-line dual-enzymatic method with gel-permeation high-performance liquid chromatography at Liposearch Skylight Biotech, Akita, Japan [6]. This method measured TG and Chol levels in each lipoprotein fraction: chylomicrons (CM, >80 nm), very-low-density lipoproteins (VLDL, 30-80 nm), low-density lipoproteins (LDL, 16-30 nm), and high-density lipoproteins (HDL, 8-16 nm). The levels of VLDL/LDL-Chol and HDL-Chol in plasma of Flox, Pla2g6^M-/-^ and Pla2g6^Hep-/-^ under chow or HFD were determined with a colorimetric method using Sigma MAK045 kit.

**Enzyme-linked Immunosorbent Assays (ELISA)**

Plasma insulin levels and leptin were determined by murine ELISA kits from Crystal Chem/Hölzel (#90080, Köln, Germany) and PeproTech (#900-K76, Hamburg, Germany), respectively. Homeostasis model assessment insulin resistance (HOMA-IR) was calculated according to: HOMA-IR = fasting insulin (μU/ml) × fasting blood glucose (mmol/l)/22.5. Concentrations of TNF-α, MIP-1α, IL-6, IL-4, IL-13, MCP-1, and KC/CXCL1 were quantified using murine standard ABTS ELISA development kits from PeproTech. The levels of lipoxin A4 in liver were determined using an ELISA kit from Cayman/Biomol (#G-UNF10044.96, Hamburg, Germany), and contents were reported as pg/mg liver. The levels of LPS in plasma (pg/ml) and liver (pg/mg liver) were determined using a mouse LPS ELISA kit from MyBioSource/Biozol (MBS040441, Hamburg, Germany).

**Histology and Immunohistochemistry (IHC)**

Formalin-fixed liver tissues were embedded in paraffin and sectioned into 4-μm slices. For histological examination, liver sections were stained with hematoxylin and eosin (H&E) according to standard protocols. Collagen deposition was assessed by staining with 0.1% direct red 80 solution (Sigma-Aldrich) dissolved in saturated picric acid. For IHC analysis, antigen retrieval was performed by heat-induced epitope retrieval in citrate buffer (pH 6.0) for 20 min. Following blocking with hydrogen peroxide, slides were blocked with either 10% normal goat serum or 2% rabbit serum. Slides were incubated overnight at 4°C with a primary antibody against Ly6G (RB6-8C5, #14-5931-85, Invitrogen, 1:150 dilution), F4/80 (SP115, ab111101, Abcam, 1:300 dilution), eosinophil cationic protein (ECP) (#PA5-79927, Invitrogen, 1:250 dilution), CD3 (SP162, #ab5690, Abcam, 1:100 dilution), CD45R (RA3-6B2, #ab64100, Abcam, 1:150 dilution), α-SMA (E184, ab32575, Abcam, 1:250 dilution) or COL1A1 (alpha-1 type 1 collagen) (E8F4L-XP, #72026, Cell Signaling, 1:100 dilution). For IHC of Ly6G, biotinylated rabbit anti-rat IgG H+L antibody (VEC-BA-4000, Biozol, Eching, Germany) and streptavidin-HRP (#70595, Abcam) were used for detection. For the rest of antibodies, goat anti-rabbit ABC (#ab64261, Abcam) and broad-spectrum (#HRP008DAB, ZytoMed) HRP/DAB kits were used for detection. Light microscopy was used to visualize stained slides with an Olympus AX 50 microscope. The stained slides were photographed with 15-20 pictures per slide using the Olympus Cell F software. The quantitative analysis of Sirius-red and IHC (+) areas was performed using ImageJ software (Fiji v1.53c). For analysis of lipid droplets in livers of HFD-fed mice, four pictures at x200 high-power-fields (HPF) were taken from each slide/mouse, and the diameters (μm) of about 100 to 500 lipid droplets in these four pictures were measured by using Olympus Cell F software. The numbers of lipid droplets in each slide/mouse were counted and grouped according to the diameter sizes of lipid droplets: > 200, 150-200, 100-150, 50-100, and 20-50 μm.

**Western blot**

Liver lysates were prepared after centrifugation at 13,000 x g for 10 min at 4°C, and their protein concentrations were quantified using a Bio-Rad protein^DC^ assay kit (Munich, Germany). Proteins at 30 or 80 μg were separated by SDS-PAGE and transferred onto PVDF membranes. After blocking with 5% milk, membranes were incubated overnight with a primary antibody against iPLA2β (D-4, sc-376,563 or T-14, sc-14,463, Santa Cruz, Heidelberg, Germany), caspase 1 (sc-56036, Santa Cruz), CD68 (#sc-20060, KP1, Santa Cruz), cPLA2α (#5249, Cell Signaling, Frankfurt, Germany), phospho-MLKL (Ser345) (#37333, D6E3G, Cell Signaling), MLKL(#37705, D6W1K, Cell Signaling), cleaved caspase 8 (Asp387) (#9429, Cell Signaling), cleaved caspase 3 (#9664, Cell Signaling), BAX (#2772, Cell Signaling), NLPR3 (#15101, Cell Signaling), ATGL (30A4) (#2439, Cell Signaling), ACSL4 (#SAB2701949, Sigma), HL (#MBS5314627, Biosource/Biozol), gp91phox/NOX2 (#611415, BD Transduction), Ly6G (#14-5931-82, eBioscience™, RB6-8C5, Invitrogen), α-SMA (ab32575, Abcam), collagen IV (ab6586, Abcam), ANGPTL3 (250808F7) (#8619-1-RR, Proteintech), ATG5 (5M5) (81803-1-RR, Proteintech ), LC3A/B (5P12) (81004-1-RR, Proteintech), and GAPDH (#2118, Cell Signaling). Following secondary antibody incubation, proteins were visualized using Luminata Forte ECL (Millipore, Darmstadt, Germany). ImageJ software was used to analyze band density for determination of target gene/GAPDH ratio.

**Gene expression**

Liver tissues were homogenized in liquid nitrogen, and RNA was isolated using the RNeasy Mini Kit (QIAGEN GmbH, Hilden, Germany), followed by genomic DNA removal with the RNase-free DNase Set (QIAGEN GmbH). Reverse transcription was performed with a FastGene Scriptase Basic cDNA Kit (NIPPON Genetics Europe, Düren, Germany). Quantitative real-time polymerase-chain-reaction (RT-qPCR) was performed on an Applied Biosystems 7500 using Applied Biosystems TaqMan® gene expression assays. All reactions were normalized to the housekeeping gene GAPDH. The relative expression of a target gene was calculated using ∆∆-Ct transformation method.

**Statistics**

Results were presented as mean ± SEM. Statistical significance were analyzed with GraphPad Prism 5.0 (GraphPad Software Inc., CA, USA) and *p* < 0.05 was considered significant. Kruskal-Wallis tests with Dunn’s selected pair post-tests were used for multiple comparisons (*, *p* < 0.05, **, *p* < 0.01, and ***, *p* < 0.001). One-tailed (§, *p* < 0.05) or two-tailed (#, *p* < 0.05, ##, *p* < 0.01, and ###, *p* < 0.001,) Mann-Whitney U tests were used for paired comparisons.

**References**

1. Otto AC, Gan-Schreier H, Zhu X, et al. Group VIA phospholipase A2 deficiency in mice chronically fed with high-fat-diet attenuates hepatic steatosis by correcting a defect of phospholipid remodeling. *Biochim Biophys Acta Mol Cell Biol Lipids*. 2019;1864(5):662-676. doi:10.1016/j.bbalip.2019.01.012.
2. Jansakun C, Chunglok W, Altamura S, et al. Myeloid- and hepatocyte-specific deletion of group VIA calcium-independent phospholipase A2 leads to dichotomous opposing phenotypes during MCD diet-induced NASH. *Biochim Biophys Acta Mol Basis Dis*. 2023;1869(1):166590. doi:10.1016/j.bbadis.2022.166590.
3. Klement L, Jansakun C, Yan B, et al. Myeloid-specific deletion of group VIA calcium-independent phospholipase A2 induces pro-inflammatory LPS response predominantly in male mice via MIP-1α activation. *Biochim Biophys Acta Mol Basis Dis*. 2024;1870(3):167016. doi:10.1016/j.bbadis.2024.167016.
4. Zhu X, Gan-Schreier H, Otto AC, et al. iPla2β deficiency in mice fed with MCD diet does not correct the defect of phospholipid remodeling but attenuates hepatocellular injury via an inhibition of lipid uptake genes. *Biochim. Biophys. Acta Mol. Cell. Biol. Lipids* 2019;1864:677-687. doi: 10.1016/j.bbalip.2019.02.003.
5. Carr TP, Andresen CJ, Rudel LL. Enzymatic determination of triglyceride, free cholesterol, and total cholesterol in tissue lipid extracts. *Clin Biochem* 1993;26(1):39-42. doi: 10.1016/0009-9120(93)90015-x.
6. Toshima G, Iwama Y, Kimura F, et al. LipoSEARCH: analytical GP-HPLC method for lipoprotein profiling and its applications. *J Biol Macromol* 2013;13:21-32. doi:10.14533/jbm.13.21
